# Shaping Durum Wheat for the Future: Gene Expression Analyses and Metabolites Profiling Support the Contribution of BCAT Genes to Drought Stress Response

**DOI:** 10.3389/fpls.2020.00891

**Published:** 2020-07-03

**Authors:** Valentina Buffagni, Filippo Vurro, Michela Janni, Mariolina Gullì, Arturo A. Keller, Nelson Marmiroli

**Affiliations:** ^1^Department of Chemistry, Life Sciences and Environmental Sustainability, University of Parma, Parma, Italy; ^2^Institute of Materials for Electronics and Magnetism (IMEM), National Research Council (CNR), Parma, Italy; ^3^Institute of Bioscience and Bioresources (IBBR), National Research Council (CNR), Bari, Italy; ^4^Bren School of Environmental Science & Management, University of California, Santa Barbara, Santa Barbara, CA, United States; ^5^CINSA Interuniversity Consortium for Environmental Sciences, Parma/Venice, Italy

**Keywords:** branched-chain aminotransferase, durum wheat, drought stress, gene expression, target metabolomics

## Abstract

Global climate change, its implications for agriculture, and the complex scenario presented by the scientific community are of worldwide concern. Drought is a major abiotic stress that can restrict plants growth and yields, thus the identification of genotypes with higher adaptability to drought stress represents one of the primary goals in breeding programs. During abiotic stress, metabolic adaptation is crucial for stress tolerance, and accumulation of specific amino acids and/or as secondary metabolites deriving from amino acid metabolism may correlate with the increased tolerance to adverse environmental conditions. This work, focused on the metabolism of branched chain-amino acids (BCAAs) in durum wheat and the role of branched-chain amino acid aminotransferases (BCATs) in stress response. The role of BCATs in plant response to drought was previously proposed for *Arabidopsis*, where the levels of BCAAs were altered at the transcriptional level under drought conditions, triggering the onset of defense response metabolism. However, in wheat the role of BCAAs as a trigger of the onset of the drought defense response has not been elucidated. A comparative genomic approach elucidated the composition of the BCAT gene family in durum wheat. Here we demonstrate a tissue and developmental stage specificity of BCATs regulation in the drought response. Moreover, a metabolites profiling was performed on two contrasting durum wheat cultivars Colosseo and Cappelli resulting in the detection of a specific pattern of metabolites accumulated among genotypes and, in particular, in an enhanced BCAAs accumulation in the tolerant cv Cappelli further supporting a role of BCAAs in the drought defense response. The results support the use of gene expression and target metabolomic in modern breeding to shape new cultivars more resilient to a changing climate.

## Introduction

Wheat is a major staple food, providing approximately 20% of daily calories and proteins uptake for 4.5 billion people. Durum wheat [*Triticum turgidum* L. *subsp. durum* (Desf.) Husn] in particular, is conventionally grown in semiarid environments (i.e., Mediterranean regions) where the optimization of the water resources is of major concern (Aprile et al., 2013). A recent study predicted a strong decline in wheat yields by 4.1 to 6.4% for each 1°C of temperature increase due to climate change (Liu et al., 2016). Consequently, while demand for wheat will increase of 60% by 2050, production might decrease by 29% as a result of environmental stresses imposed by climate change (Semenov et al., 2014). Drought, in particular, is a major challenge, thus improving drought tolerance in wheat varieties is of paramount importance for global food security. Depending on the onset time, duration and intensity of the stress, drought can reduce wheat yields up to 92% (Farooq et al., 2012; Sallam et al., 2019), which is enough to prompt the application of high-throughput tools to explore and exploit plant genomes to increase drought tolerance in wheat (Akpınar et al., 2013). In particular, knowledge of genomic variation and genetic basis of agronomically useful traits can be improved with the use of reference crop genome sequences (Scheben et al., 2016).

It is imperative to explain the molecular basis of drought stress defense response in durum wheat and to identify novel classes of genes for drought resistance which can be used in breeding programs: future world production of food depends to a large degree on the response of plants to drought stress (DS). The combination of target metabolite determination with enzymatic assays and gene-sequence and expression analyses represents a multi-disciplinary approach to identify favorable alleles that, when used as molecular markers, can speed and shape new crops for the future (Borrelli and Trono, 2016). To identify new genetic sources of drought tolerance, gene expression analysis may be performed with contrasting genotypes to identify candidate genes that are responsible for variations in a trait (Tuberosa and Salvi, 2006; Tuberosa and Pozniak, 2014; Borrelli and Trono, 2016; Yousfi et al., 2016; Kulkarni et al., 2017; Mahmood et al., 2020). Metabolomic is a complementary tool for selecting favorable genotypes on the basis of metabolomic variations. To date, a targeted metabolomic approach has been frequently used, focused on a restricted group of metabolites (Huang et al., 2018, 2019; Zhao et al., 2018). In our work, the starting point was the availability of the barley and common wheat genomes that have furnished new tools for mining useful genetic variation (Mayer et al., 2011; Xu et al., 2018). The syntenic relationship among the evolutionary related cereal genomes (Gale and Devos, 1998) has favored the use of a comparative genomic approach to discover new sources of drought resistance across cereals (Bolot et al., 2009).

Drought stress remodulates primary plant metabolism, as well as photosynthesis, including the production of carbohydrates, amino acids, and other osmolytes (Langridge et al., 2006).

In these conditions, plants avoid water loss via stomatal closure, which indirectly decreases the CO_2_ diffusion and thus the photosynthetic rate, producing a condition of metabolic constraint. When exposed to stressful conditions, plants accumulate an array of metabolites, particularly amino acids known as precursors to and constituents of proteins playing an important role in plant metabolism and development (Hayat et al., 2012). In this respect, branched-chain amino acid aminotransferases (BCATs) may play a pivotal role in triggering the onset of defense response also in wheat. In fact, the metabolism of branched-chain amino acids (BCAAs) is closely related to the primary metabolism, as isoleucine belongs to the aspartate pathway, whereas valine and leucine are derived from pyruvate (Joshi et al., 2010). In particular, BCATs play a crucial role in the metabolism of BCAAs (Binder, 2010), since they catalyze the last step of the synthesis and/or the initial step of the degradation of this class of amino acids. In plants, BCAAs are synthesized in the chloroplasts, whereas the degradation occurs in the mitochondria (Binder et al., 2007). BCATs have been previously characterized in *Arabidopsis* (Diebold et al., 2002; Urano et al., 2009; Binder, 2010; Angelovici et al., 2013), in tomato (Maloney et al., 2010), maize (Witt et al., 2012) and wheat (Bowne et al., 2012).

Several studies also indicate that a pool of these amino acids accumulates in leaves of barley (Templer et al., 2017) and wheat (Bowne et al., 2012) under water-limiting conditions. This hypothesis is supported by the fact that in *Triticeae* species, the most significant changes under drought stress can be observed in amino acids, organic acids and sugars content (Ullah et al., 2017) along with other compatible solute accumulation (Obata and Fernie, 2012). Moreover, an accumulation of BCAAs, following a regulation at the transcriptional level was observed under water-limiting conditions in *Arabidopsis* (Urano et al., 2009). The involvement of BCATs in stress response has been reported in *Arabidopsis* (Huang and Jander, 2017; Batista-Silva et al., 2019), and other plant species (Lanzinger et al., 2015; Ullah et al., 2017). Increased levels of BCAAs under drought conditions lead to higher protein degradation (Huang and Jander, 2017) or initiate a defense mechanism which stimulates the catabolic activity of BCAT and prevents the accumulation of toxic metabolites, as shown in sugarcane (Vital et al., 2017; Ali et al., 2019). In *Arabidopsis* and in other plant species both BCAAs biosynthesis and degradation occur, and these contrasting pathways must be carefully balanced to maintain homeostasis of the pool of this important group of amino acids. This is particularly true under stress conditions and critical in conditions of energy deprivation (Binder, 2010). It has also been proposed that BCAAs under conditions of energy shortage are catabolized in the TCA cycle to generate energy (Galili et al., 2016). Thus, an up-regulation of the anabolic enzyme could contribute to keep at a constant level this alternative fuel of the primary metabolism under DS conditions in wheat flag leaves.

A clear correlation between the accumulation of BCAAs under DS and their *de novo* synthesis has not been demonstrated yet, but based on the evidences in the literature (Scarpeci et al., 2016) it can be argued that the gene *BCAT3* contributes to acquire a higher tolerance as well as a good yield under DS in *Arabidopsis*. In barley, a comparative genomic approach was used to characterize the *HvBCAT-1* gene, involved in the drought defense response (Malatrasi et al., 2006).

The variation in levels observed in the pool of free amino acids correlates with the level of drought stress tolerance of the cultivars. BCAAs, in particular, increase as a consequence of the upregulation of catabolic enzymes including BCAT (Lanzinger et al., 2015). Moreover, BCAAs can work as alternative respiratory substrate under stress conditions (Araújo et al., 2011).

Nevertheless, the role of BCAAs in water-deficit stress tolerance is still unknown, as well as the molecular involvement of BCAT enzymes.

The increasing availability of the genomic sequences of many crops allows for the analysis of allelic variation within germplasm collection with the aim to discover useful alleles for subsequent breeding. In particular, the most common forms of nucleotide variations are single nucleotide polymorphisms (SNPs) and small insertions and deletions (indels), which may be significantly associated with phenotypic variations and plant adaptation to environmental constraints (Xia et al., 2017; Liu et al., 2018).

In this work, the molecular characterization of BCATs was performed in two durum wheat cultivars Cappelli and Colosseo, characterized by contrasting phenotypes for drought tolerance. Colosseo is more susceptible (Rampino et al., 2006) and Cappelli is more tolerant (Aprile et al., 2013) to drought. Taking advantage of a comparative genomic approach, this study aimed to: (i) identify the homoeologs of the chloroplast *BCAT* in Cappelli and Colosseo; (ii) characterize gene expression in response to drought stress during flowering and grain filling, as the most sensitive stages for grain set development and yield; and (iii) perform a targeted metabolic profiling employing nuclear magnetic resonance (NMR) spectroscopy.

## Materials and Methods

### Seed Materials and Germination Conditions

Seeds of Cappelli were provided by the Institute of Biosciences and Bioresources (IBBR) of Bari (Italy); Colosseo, by Council for Agricultural Research and Economics (CREA) of the Research centre for Genomics & Bioinformatics at Fiorenzuola d’Arda (Italy); Kronos, was kindly provided by Prof. Cristobal Uauy (John Innes Center, Norwich, United Kingdom); and Primadur, Simeto, Messapia, Ciccio, Creso, Saragolla, and Svevo, by the Center of Cereal Research (CREA-CER) of Foggia (Italy).

Seeds were surface-sterilized with 0.5% (v/v) sodium hypochlorite for 5 min with gentle agitation, then rinsed in distilled sterile water, and germinated on wet filter paper for 9 days at 6°C under dark conditions.

### Genomic DNA Isolation and Sequencing

Genomic isolation of genomic *TdBCAT* genes was carried out in the durum wheat cultivars Cappelli and Colosseo. Genomic DNA was extracted with the GenElute^TM^ Plant Genomic DNA Miniprep Kit (SIGMA-ALDRICH, St. Louis, MO, United States) from 100 mg of frozen leaves of Cappelli and Colosseo. DNA quality and quantity were assessed with Nanodrop 2000 (Thermo Scientific, Wilmington, DE, United States). The DNA integrity and quantity were also checked with agarose gel electrophoresis (1% agarose w/v, 1X TAE buffer, GelRed^TM^ as Nucleic Acid gel stain) by comparison to a λ genomic DNA ladder.

End-point PCR amplification of the full *TdBCAT-A* and *TdBCAT-B* gene sequences was carried out in a final volume of 20 μl, containing 10–20 ng of DNA, and 0.2 μM of each forward and reverse primers, 2X GoTaq^®^ Colorless Master Mix (Promega, Madison, WI, United States), in a TC-512 Thermal cycler (Techne, Duxford, Cambridge, United Kingdom). PCR conditions were optimized for different groups of primers (Supplementary Table S1):

• For primer pairs (PP) PP1-PP6, PCR amplification was performed with 2 min initial denaturation at 94°C followed by 35 cycles with 30 s denaturation at 94°C, 30 s annealing, and elongation at 72°C for 60 s/kb; 5 min final extension at 72°C.
• For PP7 and PP8 PCR amplifications were performed applying a touch-down PCR applying 2 min initial denaturation at 95°C followed by 10 cycles with 30 s denaturation at 94°C, 30 s annealing at 65°C with a drop −0.5°C/cycle, and elongation at 72°C for 60 s/kb, then followed by 35 cycles with 10 cycles with 30 s denaturation at 94°C, 30 s annealing, and elongation at 72°C for 60 s/kb; 5 min final extension at 72°C.

The optimized annealing temperatures for each primer pair are reported in Supplementary Table S1.

Amplicons of the expected size (Supplementary Table S1) were recovered from the agarose gel using NZYGelpure kit (NZYtech, Lisbon, Portugal) and sequenced (Eurofins Genomics, Ebersberg, Germany).

### Plant Growth and Stress Conditions

For the expression analysis during the germination phase (Experiment 1), seeds of ten durum wheat cultivars were used as experimental material: Kronos, Primadur, Simeto, Colosseo, Messapia, Ciccio, Creso, Saragolla, Svevo, and Cappelli. For the expression analysis during the reproductive stage (Experiment 2), only the two contrasting cultivars Cappelli and Colosseo were tested.

**FIGURE 1:**
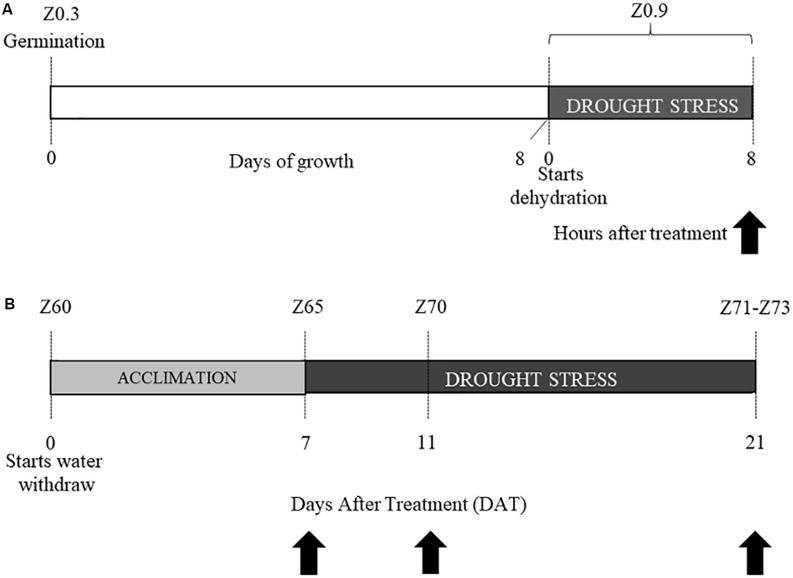
Experimental design. Experiment 1 **(A)** and experiment 2 **(B)**. Stages are indicated according to Zadoks classification as Z followed by a number. Black arrows indicate the time-points in which tissues were sampled for transcriptional analysis.

Experiment 1: After germination at 6°C, 8-days old coleoptiles (Z0.9, Zadoks scale) were dehydrated for 8 h (stress condition), or kept well-hydrated on wet paper (control condition). Ten coleoptiles from each cultivar and for each condition were sampled and immediately frozen in liquid N_2_ and kept at −80°C until RNA extraction (Figure 1A).

Experiment 2: After germination, three plants for each cultivar (Colosseo and Cappelli), stage and treatment (18 plants in total) were grown in a growth chamber (Light/Dark 16:8 h, T = 20°C, RH = 80%) under well-watered conditions until the late tilling stage (Z30, Zadoks scale) and then transplanted, with three plants per pot (4.5 L). Each pot had the same amount of soil medium mixture 8:4:1 (v/v/v) soil:peat:perlite. After transplanting, the relative Soil Water Content (RSWC) was monitored until the end of the experiment. To calculate RSWC, dry weight (DW), field capacity (FC) and the current fresh weight (FW) of soil were monitored before transplanting and during the entire experiment, as described in Aprile et al. (2013). The experimental values were entered into the formula RSWC = [(FW-DW)/(FC-DW)] × 100. Weight of the pots and plant biomass were considered for this estimation. Each pot was weighted twice a day to measure the FW, and consequently proper amounts of water were added to maintain RSWC for all the duration of the experiment. All plants were kept at 80% RSWC (control condition) until the end of the heading phase (Z60, Zadoks scale), when the water withdrawal started to reach the stress condition (30% of the RSWC) 7 days after treatment (DAT). When plants reached RSWC 30% this was considered as the acclimation to drought stress (DS). Tissue sampling for the physiological and transcriptional analysis were performed 2 h after the beginning of the light-period at three time-points: (1) 7 DAT or acclimation to DS; (2) 11 DAT; (3) and 21 DAT (Figure 1B).

For each time-point spikes and flag leaves were harvested and immediately frozen in liquid nitrogen to be kept at −80°C until RNA extraction and NMR metabolomic analysis. The leaf below the flag leaf was used for the physiological evaluation. For each cultivar and time-point, 3 plants were sampled (biological replicates; Figure 1B).

### Physiological Analyses

Physiological response to drought was investigated on three plants for each cultivar, stage and treatment during Experiment 2 by measuring leaf relative water content (RWC) and the chlorophyll content. The leaf immediately below the flag leaf was considered. Both analyses were performed immediately after the sampling for the transcriptional analysis, at the same time of the day (2 h after the start of the light period), for each time-point, condition and genotype. Leaf RWC (%) was measured in control and stressed leaves of both Cappelli and Colosseo. Five disks (Ø = 1 cm) were clipped from each leaf of 3 plants, placed in 1.5 mL tubes. Fresh weight (FW) was immediately recorded then leaf-disks were soaked in distilled water for 4 h at room temperature under constant light. The turgid leaf disks were then rapidly blotted to remove surface water and weighed to obtain turgid weight (TW). Tissues were dried in the oven at 80°C for 24 h to record the dry weight (DW). The RWC was calculated according to the formula: RWC (%) = [FW-DW)/(TW-DW] × 100 (Barrs and Weatherley, 1962).

Chlorophyll content was measured in control and stressed leaves of both Cappelli and Colosseo with a SPAD-502 Minolta (Spectrum Technologies Inc., Plainfield, IL, United States) following the manufacturer instructions. Ten measures for each leaf of 3–4 plants were recorded along the leaf length (technical replicates).

### RNA Isolation and Gene Expression Analyses by RT-qPCR

Total RNA was extracted from flag leaves or spikes of three plants with RNeasy Plant Mini Kit (Qiagen, Hilden, Germany). Plant samples were collected, pooled and homogenized in liquid nitrogen and 100 mg were used for the extraction. The total RNA was displayed on agarose gel (1% agarose w/v, 1X TAE buffer, GelRed^TM^ as Nucleic Acid gel stain) to assess its integrity. For each sample, 1 μg of total RNA was retro-transcribed into cDNA with QuantiTect Reverse Transcription Kit (Qiagen) according to the manufacturer instructions.

The expression analysis of each target gene was performed as a two-step real-time quantitative PCR (RT-qPCR). 1 μL (dilution 1:10) of cDNA was used for the analysis with a ABI PRISM 7000 Sequence Detection System (Applied Biosystems, Foster City, CA, United States). Reactions were set up in 10 μL volume with 2X PowerUp SybrGreen Master Mix (Life Technologies, Carlsbad, CA, United States) and 300 nM of gene-copy specific forward and reverse primers (Supplementary Table S1).

*TaActin* gene (AB181991, Rocchi et al., 2012) was used as house-keeping gene for normalization whereas the expression of durum wheat drought-related gene *TdDHN15.3* (AM180931.1, Rampino et al., 2006) was used to evaluate the stress conditions in the tissues. The mean Ct measured for the reference gene actin varied by less than 2.2%. All qPCR data were analyzed using the 2^−ΔΔ*Ct*^ method using control samples as calibrator (Livak and Schmittgen, 2001). The qPCR data are presented as the mean values calculated from three biological replicates with three technical replicates.

### Bioinformatic Analysis

The genomic sequence of *TdBCAT* isolated and sequenced in cv. Cappelli and cv. Colosseo were compared with the reference sequence of *T. durum* cv. Svevo^1^, using BLASTn algorithm (Altschul et al., 1990). The proximal promoter region (up to 1000 bp upstream start codon ATG) were analyzed using PlantPan 3.0 (Chow et al., 2019) considering *Oryza sativa* as a model species for *Triticeae*.

The gene topography and the putative transcription starting site were inferred using the FGENESH + (Solovyev et al., 2006) of the Softberry suites (Softberry Inc., Mount Kisco, NY, United States) starting from a similar protein HvBCAT (CAE00460.2). The predicted protein sequences were deduced and used for the following analyses. The protein localization was hypothesized exploiting the WOLF pSORT predictor (Horton et al., 2007).

Molecular evolution of BCATs was inferred by using the Maximum Likelihood method based on the Poisson correction model, on multiple alignment performed with the MUSCLE algorithm (Edgar, 2004) and the tree with the highest log likelihood was considered, (Maximum Likelihood method, 1000 Bootstrap Replications) using the Poisson model as substitution model (Zuckerkandl and Pauling, 1965). Initial tree(s) for the heuristic search were obtained automatically as described in Tamura et al. (2011) by applying Neighbor-Join and BioNJ algorithms (Saitou and Nei, 1987; Gascuel, 1997) to a matrix of pairwise distances estimated using a JTT model, and then selecting the topology with superior log likelihood value. The analysis involved 39 amino acid sequences. All positions with less than 95% site coverage were eliminated; fewer than 5% alignment gaps, missing data, and ambiguous bases were allowed at any position. The tree sketched to scale, with branch lengths measured in the number of substitutions per site. Evolutionary analyses were conducted in MEGA6 (Tamura et al., 2013).

### Metabolomic Through NMR and Data Analyses

Comparative metabolic profiling was performed on flag leaves tissues derived from the Experiment 2 (Figure 1B), collected from Cappelli and Colosseo at 7, 11, and 21 DAT both in control and drought stressed conditions. Leaves and spikes tissues previously sampled and stored at −80°C were ground to a fine powder in liquid N_2_ with a pestle and mortar, then freeze-dried at a vacuum level of 0.2 mbar and −50°C (Lio5P, 5Pascal, Milan, Italy). Samples were prepared for the NMR analysis according to Kim et al. (2010). In particular, 50 mg of ground samples were transferred into an Eppendorf tube and extracted with a mixture of 1:1 v/v CH_3_OH-d4:KH_2_PO_4_ (Acros Organics, Thermo, United States) at pH 6.0, containing 0.1% (wt/wt) sodium trimethylsilyl propionate (TSP). Samples were vortexed for 1 min and sonicated for 20 min at room temperature, then centrifuged at 17,000 × *g* for 10 min. Centrifugation was repeated until a clear supernatant was obtained. The supernatants were then transferred separately into the NMR tubes for NMR measurements. Samples were immediately processed after extraction using the following method: one-dimensional 1H-NMR spectra (three replicates for each time point, treatment and cultivar) were acquired with JEOL – ECZ600R/M3 (Joel, Japan) spectrometer at the proton frequency of 600.1723 MHz using the following parameter: 128 scans, sweep window 15 ppm, 64 k points and a calibrated 90° pulse length of 8.86 μs at 25°C using Carr-Purcell-Meiboom-Gill (CPMG) pulse sequence with a constant time CPMG delay, Trelax, of 20 ms. A 10 s relaxation delay was found to be adequate for the acquisition of quantitative data.

These parameters gave a total analysis time of 36 m 22 s. The spectra were processed and analyzed with Chenomx NMR suite 8.1 (Chenomx Inc., Edmonton, AB, Canada), zero-filing to 128 k points and line broadening 0.5 Hz22. All data were referenced to the TSP peak at 0 ppm.

Spectral matching, metabolites assignment and identification were performed with Chenomx NMR Analysis Software libraries that automatically fit the experimental spectrum to the Spectral Reference Library. The software automatically adjusts the Reference Library and allows the user to adjust peak intensity and define the exact location to determine the concentration values. Then, the intensity and position of the peak (Supplementary Table S2) were manually adjusted to generate concentrations of the selected primary metabolites detected in the plant samples.

### Statistical Analyses

Student’s *t*-test was applied to compare data of control and stressed plants for the analyses of physiological traits.

Nuclear magnetic resonance data were statistically analyzed applying the one-way homoskedastic Student’s *t*-test and standard error (SE) was calculated between replicas using Office Excel 365 ProPlus (Microsoft Corporation, Redmond, WA, United States). To overcome a recurrent limitation in metabolomic experiments which is the small number of replicates, *p*-values have been adjusted applying the corrected FDR-adjusted *p*-values for multiple comparisons following the method proposed by Benjamini and Hochberg, 1995. The application of the FDR correction to adjust the *p*-values is expected not only to reduce false positives, but also to minimize false negatives; the p.adjust function (R stat package)^2^ was used (Jafari and Ansari-Pour, 2019; Piasecka et al., 2019; Fukushima et al., 2020).

## Results

### Identification and Genomic Characterization of TdBCAT

A comparative genomic approach was utilized to identify *TdBCAT-A* and *TdBCAT-B* genes in Colosseo and Cappelli, using the barley *HvBCAT-1* (AJ574850) gene as reference. First of all, the *HvBCAT-1* matching sequences in either A and B genomes of *Triticum aestivum* were considered, on the basis of the IWGSC-CSS assembly (IWGSC, 2014). Top-score matches were used to reconstruct *in silico* the bread wheat sequences of *TaBCAT* including chromosome position, and transcript annotation (Traes), if available (Supplementary Table S3).

*TaBCAT* sequence localized on chromosomes group 4 of *T. aestivum* cv. Chinese Spring, in particular on the short arm of chromosome 4A (4AS), and on the long arm of both chromosome 4B (4BL) and 4D (4DL). This is in agreement with previously reported pericentric inversion on chromosome 4A, which caused the partial transfer of 4AL to 4AS (Devos et al., 1995) and the reported localization of *HvBCAT-1* on chromosome 4H in barley (Malatrasi et al., 2006). The chromosomal location was further verified and confirmed by investigating the durum wheat genome of Svevo^3^, matching an identical sequence on chromosomes 4A and 4B (identity >99.8%, *E*-value 0.0).

Locus-specific primer pairs allow the *in vivo* identification of the *TdBCAT-A* and *TdBCAT-B* genes in Colosseo and Cappelli. Two different alleles for *TdBCAT-A* (MK648150 and MK648149) were identified sharing 99.87% identity, while a common *TdBCAT-B* allele (MK648151) was identified in both cultivars. The analysis of *TdBCAT-A* sequence in the two cultivars showed for the Colosseo allele a 100% identity with the *–A* sequence retrieved in the Svevo reference genome. Moreover, five SNPs explaining the 0,13% variation, were found in the *TdBCAT-A* sequence. Three SNPs were located in the promoter region at 809 (T/G), 705 (T/C) and 681 (A/G) bp downstream of the ATG, and two SNPs (G/T and T/A) were observed in the first intron 218 and 219 bp downstream of the ATG, respectively. Although the presence of SNPs was retrieved only in non-coding regions of *TdBCAT-A* genes, they can impact on the gene expression thus on the phenotypic variation (Fiume et al., 2004; Webb et al., 2009; Trujillo-Moya et al., 2018). The overall conservation of *BCAT* genes is shown in the multiple sequence alignment (Supplementary Figure S1).

**FIGURE 2:**
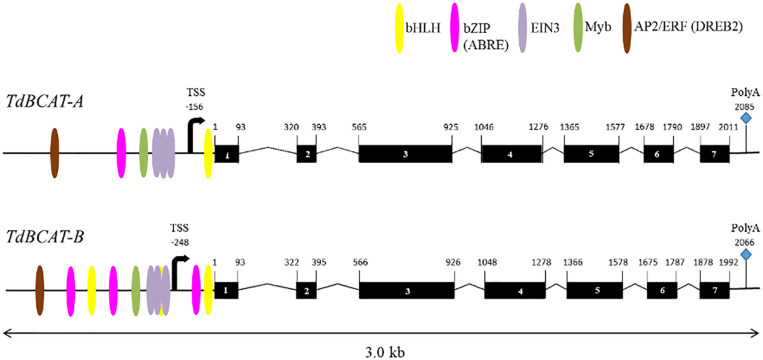
Schematic representations of TdBCAT homoeolog genes. Exons are represented as black boxes. The most relevant TFs binding site on the promoter regions are shown: Basic Helix-Loop-Helix (bHLH), Basic Leucine Zipper Domain (bZIP; ABRE), Ethylene Insensitive 3 (EIN3); Apetala2/Ethylene Responsive Factor (AP2/ERF, DREB2). The predicted sites for transcription start (TSS) and termination (PolyA) are shown.

A full list of the genes identified and their coordinates in three different wheat genome assemblies, CSS assembly (IWGSC, 2014), the TGAC v.1 (Clavijo et al., 2017) and the RefSeq v.1 (IWGSC, 2019) are reported in Supplementary Table S4. The gene’s topography predicted by the *in silico* analysis (Figure 2) confirms the 7 exons-structure previously reported for *HvBCAT-1* (Malatrasi et al., 2006). The coding sequences (CDSs) of 1200 bp-long, encoded a protein of 399 amino acids for both *TdBCAT-A* and *TdBCAT-B*. A transcriptional start site (TSS) was predicted at 156 bp and 248 bp upstream the starting codon in *TdBCAT-A* and *TdBCAT-B*, respectively, and a PolyA site was retrieved 2085 bp and 2066 bp downstream of the ATG.

### Promoter Analysis

The promoter sequences of *TdBCAT-A and TdBCAT-B* were analyzed with Plant PAN 3.0 database (Chow et al., 2019). The complete list of the Transcription factors binding sites (TFBS) identified is reported in Supplementary Table S6. Here the TFBS related to drought stress are reported in Table 1.

**TABLE 1:** List of some regulatory elements in TdBCAT promoters.

TF family	TF(s)	Position^(a)^		Binding sequence		TFBS ID^(b)^
			
		*TdBCAT-A*	*TdBCAT-B*	*TdBCAT-A*	*TdBCAT-B*	
AP2/ERF	DREB2	−758 (+)	−817 (−)	(+) gCACCGaca	(−) gggCGGTGa	TFmatrixID_0066
bHLH			−584 (±)		(+) GCTCGttc	TFmatrixID_0174;
			−218 (±)		(+) aacACTTGc	TFmatrixID_0176
			−20 (±)		(+) GCCCGggc	
		−12 (±)	−12 (±)	(+) GTACGtgc	(+) GTACGtgc	
bZIP	ABF3		−673 (±)	(+) CCACGggt	(+) AGACGtat	TFmatrixID_0193
		−455 (±)	−507 (±)		(+) CCACGggt	
			−93 (±)		(+) AAACGtat	
EIN3	EIN3	−280 (±)	−329 (±)	(+) taATGCAtgt	(+) taATGCAtgt	TFmatrixID_0256
		−250 (±)	−299 (±)	(+) caATGCAtgt	(+) caATGCAtgt	
		−229 (±)	−265 (±)	(+) caATGCAtgt	(+) caATGCAtgt	
MyB			−709 (±)		(+) atAATATttt	TFmatrixID_0334;
			−683 (±)		(+) atAATATtat	TFmatrixID_0320;
		−313 (±)	−372 (±)	(+) caaATATCt	(+) caaATATCt	TFmatrixID_0357

*The relative transcription factors (TFs) predicted to bind the Transcription Factors Binding Sites (TFBS) are indicated. (a) Positions consider as + 1 the A of the start codon (ATG) of the corresponding gene. In brackets the position on the plus(+) and/or minus(−) strand; (b) TFBS ID from PlantPan3.0 database.*

One Dehydration-Responsive Element-Binding (DREB) is predicted in either A and B gene promoters as targeted by DREB2 subunits of the AP2/ERF family, which are key regulators in many abiotic stress responses including drought, high salinity and heat stress (Zhang et al., 2009; Yousfi et al., 2016; Xie et al., 2019). The two genes show a different number of Abscisic-acid Responsive Elements (ABRE) targeted by ABF3 and several TFs of the bHLH family. In particular, *TdBCAT-B* has the highest number of ABREs elements as compared to *TdBCAT-A.*

A conserved triplet of motifs targeted by EIN3 was identified in both *-A* and *-B* genes. EIN3 is a TF that regulates primary plant response to ethylene, a well-known stress-response hormone and regulator of plant growth and development (Khan et al., 2017).

### Phylogenetic Analysis and Subcellular Localization of BCATs

The BLASTp analysis of the protein sequences identified TdBCAT proteins as a chloroplastic-like Branched-Chain Amino Acid Amino-Transferase, since the first 100 score matched only to chloroplast or to chloroplastic-like proteins, in accordance with the WoLFPSORT prediction (score: *k-*nearest neighbors = 14) (Horton et al., 2007). Only three sequences showing a 66% of identity were annotated as mitochondrial BCATs: BCAT1 of *Morus notabilis* (XP_024029919.1), BCAT1 of *Ziziphus jujuba* (XP_015885109.1) and BCAT1 of *Momordica charantia* (XP_022132131.1).

A multiple alignment was performed on 39 sequences, including previously isolated BCATs and all the AtBCAT isoforms of *Arabidopsis thaliana* described as mitochondrial, chloroplastic and cytosolic.

**FIGURE 3:**
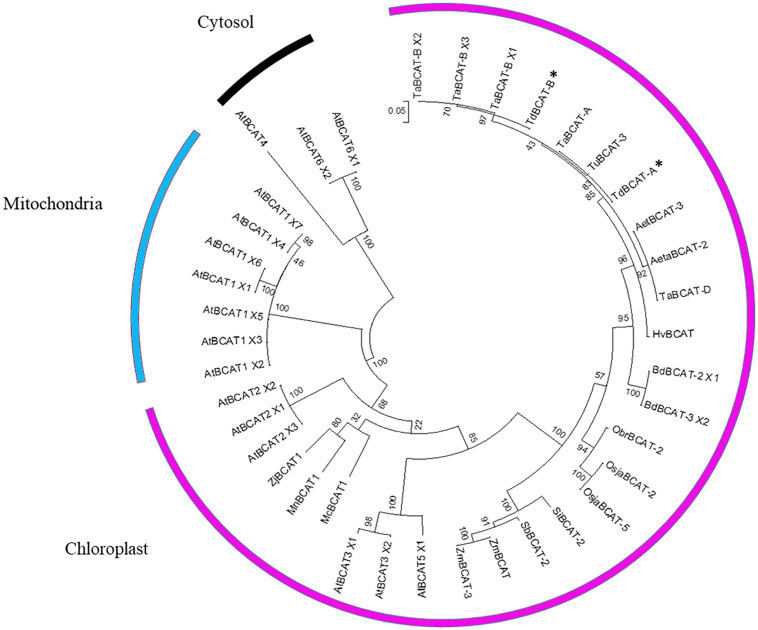
Phylogenetic tree. The TdBCAT-A and –B deduced protein sequences were used to design the phylogenetic tree. The tree was obtained by Maximum Likelihood method using MEGA 6 (Tamura et al., 2013); the highest log likelihood (–3956.0220) is shown. Asterisks indicate the newly isolated TdBCATs (*). TuBCAT-3 (*Triticum urartu*, EMS62409.1), HvBCAT (*Hordeum vulgare* CAE00460.2), Aeta BCAT-2 (*Aegilops tauschii*, XP_020159191.1), AetBCAT-3 (*Aegilops tauschii*, XP 020173856.1), BdBCAT-2 × 1 (*Brachypodium distachyon*, XP_003558433.1), BdBCAT-3_X2 (*B. distachyon*, XP_010228932.1), OsjaBCAT-2 (*Oryza sativa* Japonica group, XP_015630708.1), OsjaBCAT-5 (*O. sativa* Japonica group, ABF94786.1), ObrBCAT-2 (*Oryza brachyantha*, XP_006651192.1), SiBCAT-2 (*Setaria italica*, XP_004985120.1), SbBCAT-2 (*Sorghum bicolor*, XP_021317757.1), ZmBCAT (*Zea mays*, XP_008674369.1), ZmBCAT-3 (*Z. mays*, PWZ58251.1). The only three BCATs descripted as mitochondrial are MnBCAT1 (*Morus notabilis*, XP_024029919.1), ZjBCAT1 (*Ziziphus jujuba*, XP_015885109.1) and McBCAT1 (*Momordica charantia*, XP_022132131.1). Sequences retrieved from Ensembl Plant database are the *Triticum aestivum* TaBCAT-A (TraesCS4A01G059800.1), TaBCAT-B_X1, X2 and X3 (TraesCS4B01G235400.1, 0.2, 0.3), TaBCAT-D (TraesCS4D01G236800.1), and the *Arabidopsis thaliana* AtBCAT1_X1, X2, X3, X4, X5, X6, X7 (AT1G10060.1, 0.2, 0.3, 0.4, 0.5, 0.6, 0.7), AtBCAT2_X1, X2, X3 (AT1G10070.1, 0.2, 0.3), AtBCAT3_X1, X2 (AT3G49680.1, 0.2), AtBCAT5_X1, X2 (AT5G65780.1, 0.2), AtBCAT6_X1, X2 (AT1G50110.1, 0.2), AtBCAT4 (AT3G19710).

The clustering (Figure 3) placed TdBCAT-A and TdBCAT-B in the same subtree with three chloroplastic BCATs of *Arabidopsis* (AtBCAT-2, AtBCAT-3, AtBCAT-5) (Diebold et al., 2002). As expected, all the cytosolic and the mitochondrial BCATs clustered in two distinct subtrees, although the mitochondrial BCATs fell into the chloroplast subtree, indicating a possible mis-annotation of these sequences. The phylogenetic analysis reveals high similarity between the isolated TdBCATs and the *Triticum urartu* BCAT; moreover, high identity was observed within the BCATs in the *Triticeae* tribe and in general between *Gramineae*, followed in order of similarity by *Brachypodium distachyon*, *O. sativa, Setaria italica, Sorghum bicolor*, and finally *Zea mays.*

### Physiological Traits Analyses

The effects of drought stress on Cappelli and Colosseo, were evaluated in Experiment 2 considering the leaf immediately below the flag leaf. Three-time points were considered: 7, 11, and 21 DAT.

**FIGURE 4:**
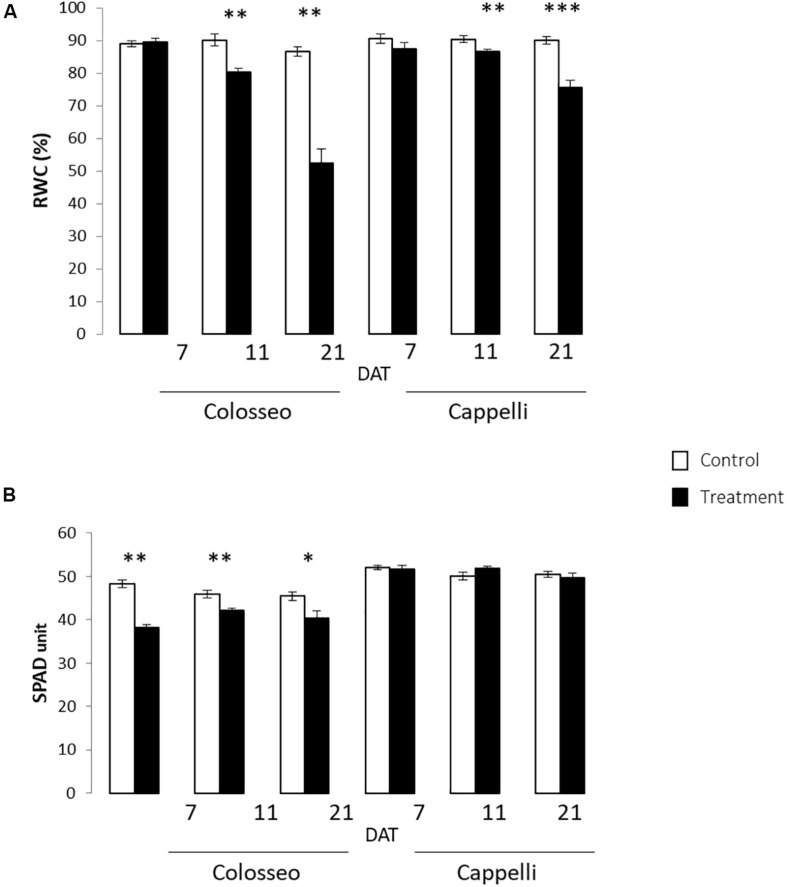
Physiological analyses. Were performed on leaves of Colosseo and Cappelli at three time-points: 7, 11, and 21 days after treatment (DAT) in control (white) and treated (black) plants. Three plants each time point for each treatment have been considered. **(A)** Relative Water Content (RWC) values are presented as the mean (±SE) of ten leaf disks. **(B)** Chlorophyll content values are presented as the mean (±SE) of ten measures for 3–4 leaves. Significantly different values are indicated with asterisks (^∗∗∗^*p-value* ≤ 0.001; ^∗∗^*p-value* ≤ 0.01; ^∗^*p-value* ≤ 0.05 evaluated with Student’s *t*-test.).

A significant progressive reduction of the RWC starting from 11 DAT (Z65) was observed in both cultivars (*p-value* ≤ 0.01), more consistent in the sensitive genotype Colosseo confirming its higher susceptibility to drought stress. Two weeks after the stress imposition the RWC was 50% in Colosseo compared with the 71% detected in Cappelli (Figure 4A).

The relative chlorophyll content measured with SPAD confirmed the overall health status of the plants, and showed in Colosseo stronger significant reduction of chlorophyll content in particular at 7 DAT (*p-value* ≤ 0.01) (Figure 4B).

The physiological analyses confirmed the higher DS tolerance of Cappelli, which, in drought conditions, maintains a higher RWC in leaves and a higher photosynthetic capacity.

### Gene Expression Analyses

**FIGURE 5:**
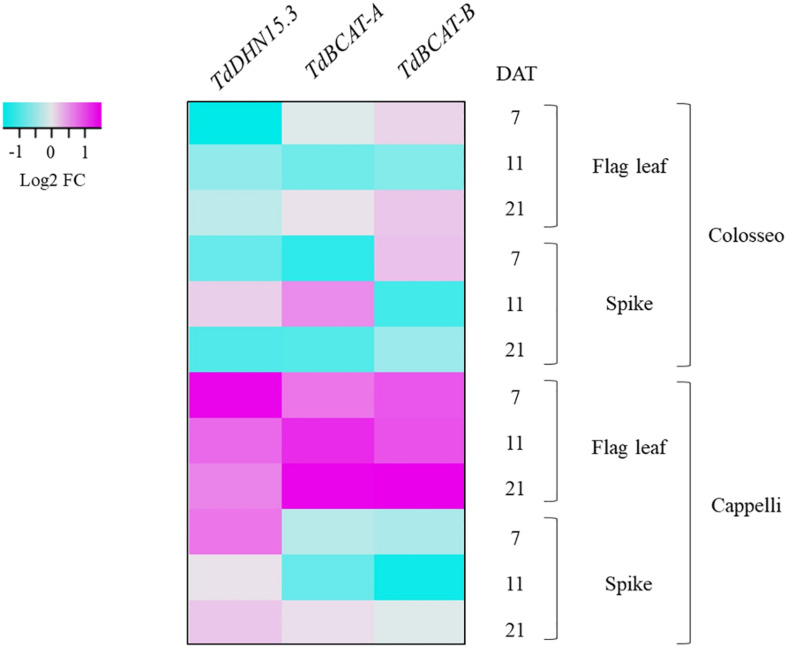
Heat-map showing the relative expression of *TdBCAT-A*, *TdBCAT-B*, and *TdDHN15.3* in response to drought stress in the flag leaf and spike. Three different time-points (7, 11, and 21 days after treatment, DAT) in the reproductive stage in three plants for each treatment of drought-sensitive (Colosseo) and drought-tolerant (Cappelli) durum wheat cultivars were considered. Data are expressed as log2 Fold-change (FC).

The modulation of *TdBCAT* genes in response to DS was monitored under water-limiting conditions in two relevant phases of durum wheat development: germination (Experiment 1) and reproductive stages (Experiment 2). The dehydrin gene *TdDHN15.3* (AM180931.1), was used as control since is known to be strongly up-regulated in dehydrated durum wheat coleoptiles and in barley flag leaves under terminal drought (Rampino et al., 2006; Karami et al., 2013). This set of experiments showed strong homoeologs and cultivar-specificity in the expression profile of the newly identified *TdBCAT* genes (Figure 5).

During germination *TdBCAT-A* is not significantly modulated in response to DS in all cultivars tested, while *TdBCAT-B* is up-regulated, though moderately, in most of the cultivars tested, with the exception of Ciccio and Svevo in which the down-regulation is still below 2-fold as compared to controls (Table 2). In Cappelli, *TdBCAT-B* expression increases about 2-fold in dehydrated coleoptiles. *TdDHN15.3* shows the highest up-regulation from 300-fold to almost 1946-fold, depending on the cultivar (Table 2). This is consistent with the observation reported by Rampino et al. (2006) in durum wheat cultivars exposed to severe drought stress conditions, which observed comparable expression levels of many dehydrin genes, including *TdDHN15.3.*

**TABLE 2:** Relative expression of TdDHN15.3 and TdBCAT genes in coleoptiles of dehydrated plantlets.

	**Creso**	**Kronos**	**Messapia**	**Primadur**	**Ciccio**	**Simeto**	**Saragolla**	**Svevo**	**Colosseo**	**Cappelli**
*TdDHN15.3*	317.4	1, 946.50	406.37	1, 016.93	421.68	661.68	1, 072.43	1, 930.82	789.61	301.29
*TdBCAT-A*	0.84	0.92	0.84	1.09	0.46	0.87	1.02	0.69	1.78	1.33
*TdBCAT-B*	1.24	1.44	2.35	1.84	0.67	1.18	1.42	0.9	1.47	2.43

*Ten durum wheat cultivars were considered. Values represent fold-change (FC) mean values of dehydrated samples in comparison with the regularly irrigated once.*

In plants subjected to a prolonged stress from the end of heading phase to the early grain-filling stage (Experiment 2) *TdDHN15.3* showed a strong up-regulation at all-time points, mainly in Cappelli (Supplementary Table S5). In particular, the highest fold change (FC) values were observed at 7 DAT both in flag leaves (FC 19.84) and in spikes (FC 8.63), while lower values were observed at 21 DAT (early post-anthesis) n leaves (FC 7.73) and spike (FC 4.63). Our data agree with the results of Karami et al. (2013) of a remarkable higher induction of this gene in barley flag leaf, in tolerant genotypes (Supplementary Table S5). On the other hand, in Colosseo, *TdDHN15.3* expression is consistently lower as compared to Cappelli, with the maximum level of induction in spike tissues at 11 DAT (FC 4.40), while in flag leaves the highest over-expression was recorded at 21 DAT (FC 2.55, Supplementary Table S5).

*TdBCAT-A* and *TdBCAT-B* were up-regulated in the flag leaves under DS, reaching the maximum in Cappelli at 21 DAT, with FC 13.5 and 16.37, respectively (Figure 5 and Supplementary Table S5). In Colosseo, the up-regulation was less consistent and followed a different trend: in flag leaves both genes had similar level of expression at 7 and 21 DAT (FC 2.0–2.4 and FC 3.21–3.54), with no relevant changes at 11 DAT; in spikes *TdBCAT-A* was induced at 11 DAT (FC 4.62), while *TdBCAT-B* was progressively down regulated from 7 to 21 DAT (FC from 3.61 to 1.47, Figure 5 and Supplementary Table S5). In Cappelli, both genes were strongly over-expressed in flag leaves, supporting the hypothesis of a higher relevance of the gene expression of this gene in leaves than in spikes under drought.

Moreover, these data highlight strong tissue specificity in the expression of *TdDHN15.3* in response to drought. In the stressed coleoptiles the highest expression was observed in the cultivar Kronos (FC 1946.50 Table 2), while in Cappelli the maximum of expression was observed in flag leaves, even if with lower extent (FC 20) (Supplementary Table S5 and Figure 5).

### Metabolic Characterization of Drought Stress Response

Plant samples from each genotype collected at 7, 11, and 21 DAT were analyzed using a targeted metabolomic approach by selecting 12 metabolites among those known to be affected by drought. These metabolites are distributed into three classes: amino acids (with a focus on BCAAs), organic acids and sugars. During DS the intracellular osmotic potential is decreased by accumulating compatible solutes such as proline (Pro), Ɣ-aminobutyric acid (GABA) and glutamate (Hildebrandt, 2018; Fàbregas and Fernie, 2019; Kang et al., 2019). Moreover, the production of sucrose, and malic acid and glutamic acid are promoted during drought stress (Marček et al., 2019).

Our results showed that Pro synthesis is increased during drought, but new insights were found regarding differences in the accumulation of other metabolites between the two durum wheat varieties.

**FIGURE 6:**
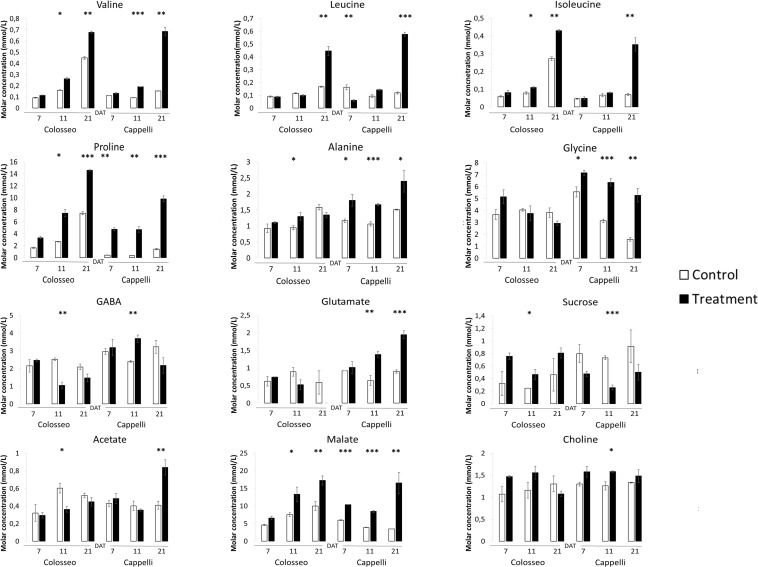
Targeted NMR metabolite profiling. The analyses were conducted on Cappelli and Colosseo leaves at three time-points: 7, 11, and 21 days after treatment (DAT) in control (white) and treated (black) plants. Three plants have been considered for the analyses. Values expressed as molar concentration (mmol/L) represent the mean (± SE) of three independent samplings. Significantly different values are labeled with asterisks (^∗^FDR-adjusted *p*-value ≤ 0.05; ^∗∗^FDR-adjusted *p*-value ≤ 0.01; ^∗∗∗^FDR-adjusted *p*-value ≤ 0.001 evaluated with Student’s *t*-test adjusted to control the false discovery rate (FDR).

Branched chain-amino acids are positively accumulated during drought stress in durum wheat. Leucine, isoleucine, and valine, increased significantly under stress conditions in both Colosseo and Cappelli with a maximum at 21 DAT (Figure 6) which correlates with the expression level of both *TdBCAT-A* and *B* in Cappelli. Analyses of the level of BCAAs have already demonstrated that valine were present at higher levels than isoleucine and leucine in stems, leaves, and flowers (Maloney et al., 2010; Kochevenko et al., 2012).

A subset of metabolites, including three amino acids (alanine, glycine), choline and glutamate and three organic acids (GABA, malate, and acetate) increased only in Cappelli which, on the contrary, exhibited a lower accumulation of sucrose. In Colosseo, drought stress induced the higher accumulation of sucrose during DS (Figure 6).

In Cappelli, the content of Pro was positively regulated during the stress with no increase at 11 DAT and a maximum at 21 DAT (Figure 6), while Colosseo showed a progressive increase reaching higher content at 21 DAT. In addition, the basal levels of Pro in the non-stressed Colosseo plants were higher than in Cappelli.

**FIGURE 7:**
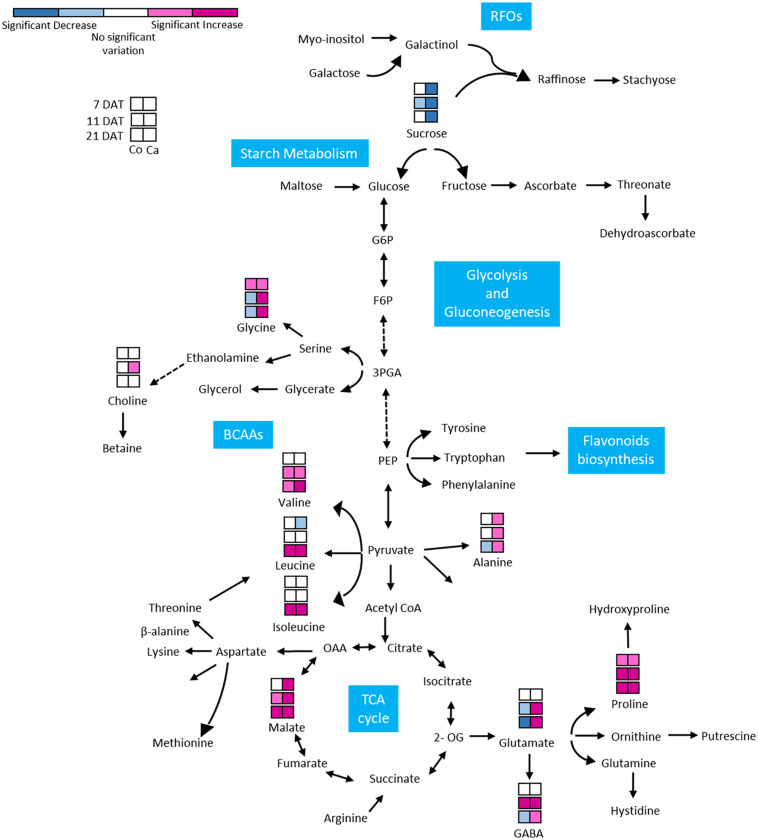
Metabolic response of Colosseo (Co) and Cappelli (Ca) plants to drought. Boxes represent the three time points analyzed (7, 11, and 21 DAT). Colors depict the relative accumulation levels of the analyzed metabolites in stressed samples as compared to the control. Metabolites with magenta and pink boxes denote significant increases while sky blue or dark blue boxes denote significant decreases. White boxes indicate no significant change. The level of significance was set at *p-value* ≤ 0.05. BCAAs, branched-chain aminoacids; RFOs, raffinose family oligosaccharides.

GABA, as part of the compatible osmolytes, was upregulated in the early phases of the drought response in Cappelli but was downregulated in Colosseo, confirming the hypothesis of an involvement of GABA in increasing drought tolerance in Cappelli, acting as ROS scavenger (Hildebrandt, 2018). Among the genotypes, the organic acids content was higher in the case of malate, which was accumulated in Colosseo (at 11 and at 21 DAT) and at all-time points in Cappelli. Acetate was significantly accumulated only in Cappelli at 21 DAT. The content of choline, the precursor of glycine-betaine slightly increased in early phases of the defense response in Cappelli (11 DAT); choline is involved in the defense response correlated with the osmoregulation process (Figure 6). A summary of the metabolic responses of Cappelli and Colosseo to drought is reported in Figure 7.

## Discussion

This paper describes the genomic characterization of the *TdBCAT*s in *T. durum* obtained through a combination of *in silico* and *in vivo* analyses that also led to the cellular localization of the *TdBCATs*. As previously reported for other plant species, the N-terminal extensions of *TdBCAT* genes targeted the protein in chloroplasts in durum wheat, suggesting a specialized enzymatic activity to complete the synthesis of either Val, Leu, and Ile. The regulation of the homeologs A and B alleles in response to DS in leaves and spikes, suggests a tissue specific function, considering their higher expression in leaves but lower in reproductive organs. In addition, a strong cultivar specificity was observed, with these transcripts more abundant in Cappelli (tolerant) than in Colosseo (susceptible). This expression pattern during drought may reflect a higher demand for amino acids in vegetative organs to counteract the effects of water deprivation.

The distinct localization patterns and the drought specific over-expression of *TdBCATs* was also confirmed by the expression of *TdBCAT-A* and -*B* in coleoptiles in eight (Creso, Kronos, Messapia, Primadur, Simeto, Saragolla, and Cappelli) out of ten cultivars tested (excluding Svevo and Ciccio) and demonstrated that BCATs participate in early phases of the DS defense response and an extremely low expression at basal levels in normal growth conditions.

The targeted NMR analysis of the metabolomic alteration of leaves in both Cappelli and Colosseo (Figure 7) indicated important alteration to the levels of several metabolites. Previous studies linked the involvement of several defense mechanisms, such as ROS balance, and signaling pathways with increased levels of metabolites in different plant species (Urano et al., 2009; Kochevenko et al., 2012; Pires et al., 2016; Todaka et al., 2017; Fàbregas and Fernie, 2019; Fukushima et al., 2020). In this study the metabolomic analysis was targeted to define the contrasting metabolic changes in leaves of two cultivars with different tolerance to DS and to search for correlations between the changes in metabolites classes and levels and the regulation of the newly identified *TdBCAT* genes.

An enhanced level of amino acids have been found in several plant species as *Arabidopsis*, wheat, soybean, bean, rice and chickpea in DS conditions (Sassi et al., 2010; Silvente et al., 2012; Rahman et al., 2017; Todaka et al., 2017; Hildebrandt, 2018; Batista-Silva et al., 2019; Kang et al., 2019). As expected, BCAAs (Leu, Ile, and Val) levels increased under water deficit at higher extent in the tolerant Cappelli and the increase seems regulated at a transcriptional level with a dehydration inducible specificity.

This is also consistent with the presence in the *TdBCAT-A* and *–B* promoters of typical ABRE motifs. It is well established that during abiotic stress low abundant amino acids like BCAAs are not synthesized but they accumulate due to an increased protein turnover in conditions of carbohydrate starvation (dehydration, salt stress, darkness) and then are degraded (Hildebrandt, 2018). Similarly, the activities of some enzymes related to the catabolism of BCAAs showed rapid increase in response to abiotic stresses, and therefore it is reasonable to assume an important role in the metabolism of BCAAs under stress (Mata et al., 2016) possibly due to the onset of BCAAs catabolism in the tolerance mechanism as suggested also by Mata et al. (2016). Moreover, it has been proposed that BCAAs under energy shortage are catabolized by the TCA cycle to generate energy (Galili et al., 2016), thus an up-regulation of the anabolic enzyme could contribute to keep at constant level this alternative fuel for the primary metabolism under DS condition in wheat flag leaves.

Proline (Pro) plays a pivotal role in mitigating drought stress by reducing ROS level and by preserving the integrity of plant membrane and proteins (Hayat et al., 2012) and its accumulation under stress has been associated with drought tolerance in many plants (Kishor et al., 2005). In a previous study, it was observed that Pro was highly accumulated in the leaves of a drought tolerant wheat cultivar at a higher concentration than in a sensitive one (Kang et al., 2019). In this analyses, a higher accumulation of Pro in leaves of the susceptible cv Colosseo was observed contrasting with previous result (Kang et al., 2019).

GABA, showed a higher accumulation in Cappelli at 21 DAT than in Colosseo where a strong reduction during the stress was observed. Glutamate, as expected, showed a similar trend since it is a precursor of GABA (Marček et al., 2019), and is accumulated in Cappelli while its content strongly decreases in Colosseo during drought stress, as previously observed in another drought sensitive wheat genotype (Michaletti et al., 2018).

Finally, the accumulation of compounds as GABA, malate and amino acids may reflect disruption of export of these compounds or their precursors from source to sink under stress conditions, since both organic acids and amino acids are common constituents of sap exudates in diverse plant species (Bialczyk and Lechowski, 1992; Hildebrandt, 2018).

In this work we observed that the choline accumulation was significantly enhanced in the early phases of the response to DS (11 DAT) in Cappelli (Gou et al., 2015), supporting for this variety increased capacity of osmoregulation during drought response and that the accumulation of compatible osmolytes such as glycinebetaine (GB) and choline is significantly involved in osmotic adjustment and protection of essential biomolecules under various environmental stresses including DS (Ashraf and Foolad, 2007; Ashraf et al., 2011).

Sugars are known to play a pivotal role as signaling molecules of many metabolic networks in plants. Drought stress is known to disrupt carbohydrate metabolism and to decrease sucrose level in leaves, presumably due to an induced increase of invertase activity (Ruan et al., 2010; Gagné-Bourque et al., 2016), hampering the rate of sucrose export to the sink organs. This hypothesis was confirmed because sugar accumulated mostly in the susceptible Colosseo, and was decreased strongly in the tolerant Cappelli. The lower abundance of sugars and the higher amount of BCAAs observed is consistent with the severity of DS imposed (Fàbregas and Fernie, 2019).

The abundance of organic acids in the drought tolerant Cappelli was increased by DS supporting its greater capacity in regulating drought stress. The relative abundance of malate correlates more with environmental factors than with water availability, and may reflect the enhancement of a malate-oxaloacetate transport to prevent over-reduction of photosynthetic electron chain components, leading to the accumulation of malate in vacuoles (Scheibe, 2004). The ability of C3 plants to store malate was likely to reflect genetic variation in key pathways, being more evident in a susceptible cultivar.

Also, the content of acetate increased specifically in Cappelli at 21 DAT. Its role seems to be as an initial factor that promotes and connects fundamental metabolism, epigenetic regulation and hormone signaling, ultimately leading to the plant drought tolerance (Kim et al., 2017).

The metabolic profiling allowed for comprehensive analyses of a range of metabolites that have great value in both phenotyping and plant genotyping. In fact, a set of water stress related metabolic biomarkers for wheat was identified as differently accumulated in contrasting cultivars opening new perspectives to select superior performing lines in breeding programs, employing targeted metabolomic as a fast and low-cost diagnostic tool.

## Data Availability Statement

The data set generated for this study can be found in the Genomic sequences are available on NCBI repository. NMR data are available upon request to the authors, and will be soon available in MetaboLights (http://www.ebi.ac.uk/metabolights/).

## Author Contributions

MJ, VB, MG, and NM conceived and designed the experiments. VB performed genomic and gene expression experiments and analyzed the data. FV performed the NMR analyses and analyzed the data under the supervision of AK. NM and MJ coordinated the entire project and preparation of the manuscript. All authors contributed to the article and approved the submitted version.

## Conflict of Interest

The authors declare that the research was conducted in the absence of any commercial or financial relationships that could be construed as a potential conflict of interest.

## Footnotes

1 https://d-data.interomics.eu/blast/nucleotide/nucleotide

2 http://www.R-project.org

3 https://d-data.interomics.eu/
