# Supplementary material for: Shaping Durum Wheat for the Future: Gene Expression Analyses and Metabolites Profiling Support the Contribution of BCAT Genes to Drought Stress Response
Source: Front Plant Sci. 2020 Jul 3;11:891. doi: 10.3389/fpls.2020.00891 (PMC7350509; doi:10.3389/fpls.2020.00891)
Supplement: Supplementary file 1 [file Data_Sheet_1.docx]

Supplementary Material

Shaping durum wheat for the future: gene expression analyses and metabolites profiling support the contribution of BCAT genes to drought stress response

Buffagni V^1^., Vurro F^3^., Janni M*^2,3^., Gulli M*^1^, Keller A.A., ^4^Marmiroli N^1,5^.

^1^Department of Chemistry, Life Sciences and Environmental Sustainability, University of Parma, Parco Area delle Scienze, 11/A, 43124 Parma, Italy

^2^Institute of Bioscience and Bioresources (IBBR), National Research Council (CNR), Via Amendola 165/A, 70126 Bari, Italy

^3^Institute of Materials for Electronics and Magnetism (IMEM), National Research Council (CNR), Parco Area delle Scienze 37/A, 43124 Parma

^4^Bren School of Environmental Science & Management, University of California, Santa Barbara, CA 93106, USA

^5^ CINSA Interuniversity Consortium for Environmental Sciences, Parma/ Venice, Italy

*** Correspondence:** [michela.janni@ibbr.cnr.it](mailto:michela.janni@ibbr.cnr.it), mariolina.gulli@unipr.it

**Supplementary Table S1 Primers pairs list.** List of primer pairs used for the isolation of *TdBCAT* gene sequences (PP1-PP8) and the expression analysis by RTqPCR (PP9-PP12). For each primer pair, the target gene, the 5’-3’ sequence, the optimized annealing temperature, and the amplicon size are reported.

| **Primer pair** | **Target gene** | **5'-3' sequence** | **Annealing temperature**  **(°C)** | **Amplicon length ^a^**  **(bp)** |
| --- | --- | --- | --- | --- |
| PP1 | *TdBCAT-A* | For- GGTCAATTGCCATCTCCGT | 58 | 1093 |
|  |  | Rev- GACGTACATGTAGTCGGTCG |  |  |
| PP2 | *TdBCAT-A* | For- GTGGAAGTCGTCGCAGCC | 61 | 1202 |
|  |  | Rev- GAGCTCGATGATGCTCCGA |  |  |
| PP3 | *TdBCAT-A* | For- CTCAAGGCGCAGATGGAC | 58 | 940 |
|  |  | Rev- TTACAAGCCTCACTCTCAGCA |  |  |
| PP4 | *TdBCAT-B* | For- CTCCACGACCTATATAGAAACCTC | 58 | 1289 |
|  |  | Rev- CGGCCTGATGTACAATGCT |  |  |
| PP5 | *TdBCAT-B* | For- GTGGAAGTCGTCGCAGCC | 62 | 1201 |
|  |  | Rev- GAGCTCGATGATGCTCCTG |  |  |
| PP6 | *TdBCAT-B* | For- CTCAAGGCGCAGATGGAC | 58 | 888 |
|  |  | Rev- CTAGCTAGGCACCATAGAGAA |  |  |
| PP7 | *TdBCAT-A* | For- GGTAGTGGCCGACGAAGG | 56 ^b^ | 1661 |
|  |  | Rev- CAGACGACAGCACAGCCAT |  |  |
| PP8 | *TdBCAT-B* | For- GCCTTGACACGACGACTTTC | 56 ^b^ | 899 |
|  |  | Rev- CAGACGACAGCACAGCCAT |  |  |
| PP9 | *TdBCAT-A* | For- GACAAGGTACGAGTTCAGGACC | 60 | 110 |
|  |  | Rev- CGACCGTCCATCCCTTCTTG |  |  |
| PP10 | *TdBCAT-B* | For- ACAAGGTACGAGTTCAGGGCT | 60 | 109 |
|  |  | Rev- TAACCGTCCATCCTTTCTTGTCCT |  |  |
| PP11 | *TdActin* | For- CTTGTATGCCAGCGGTCGAA | 60 | 173 |
|  |  | Rev- TGAGGAAGCGTGTATCCCTCG |  |  |
| PP12 | *TdDHN15.3* | For- GGAGGAGGAAGAAGGGCATCA | 60 | 116 |
|  |  | Rev- CATCCCTGCCGTATGACCTTG |  |  |

*^a^ Amplicon length of the corresponding PCR product using genomic DNA as template.*

*^b^ Final annealing temperature of the touch-down PCR.*

**Supplementary Table S2**: Metabolites identified with their Kyoto Encyclopedia of Genes and Genomes identifier number (KEGG ID/PubChem CID) in the 600 MHz Spectrum at pH 6.0 (Phosphate Buffer). ^1^H chemical shifts are referred to TSP as internal standard (δ = 0.00 ppm). Abbreviations are reported (s, singlet; d, doublet; dd, double of doublets; t, triplet; q, quartet; m, multiplet)


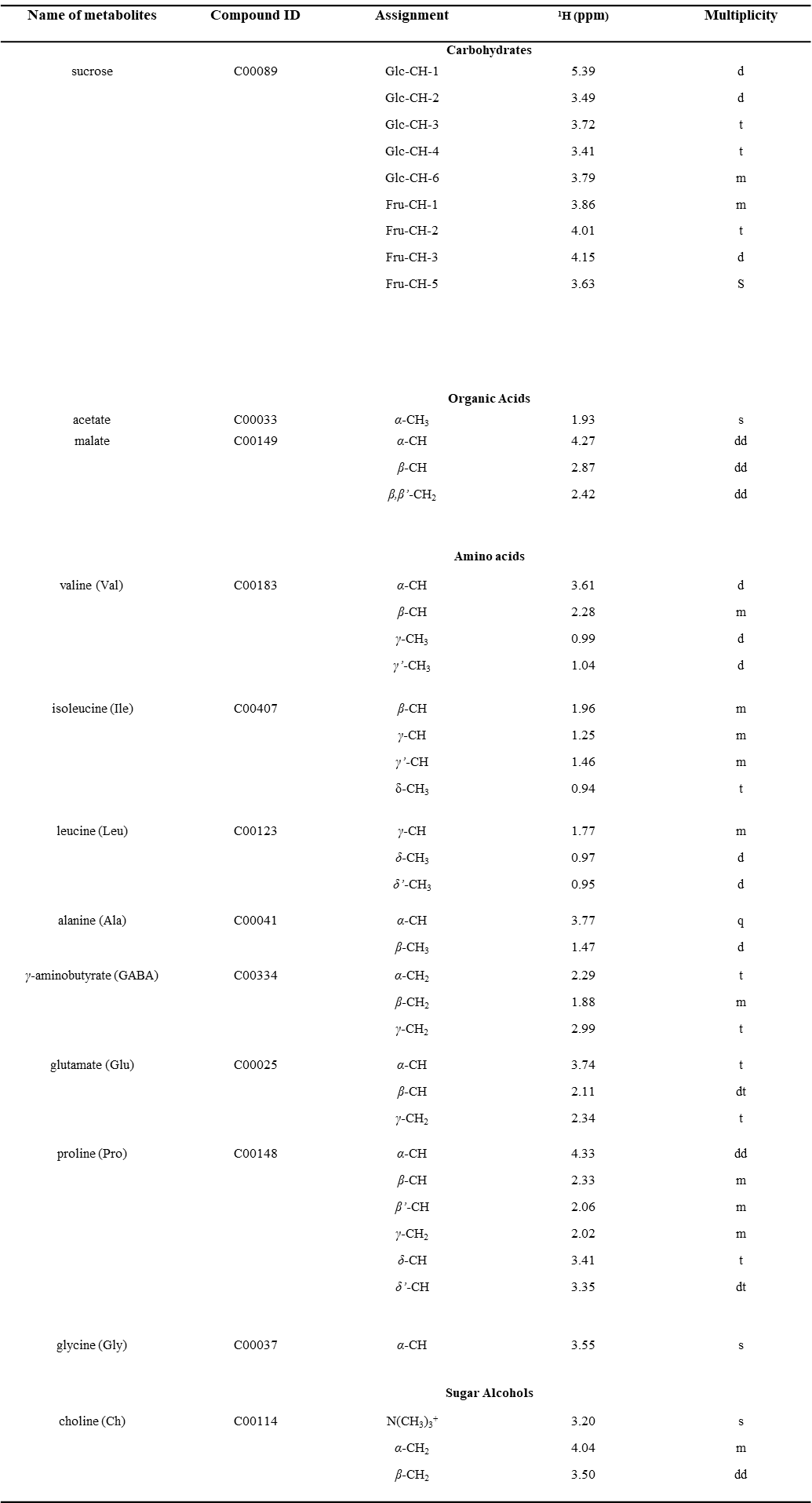


**Supplementary Table S3** Summary of the *in silico* analysis on EnsemblPlants database (IWGSC CSS assembly) for the gene *BCAT*. The genomic location, the identification (Traes) of the overlapping genes, the % of identity between the query *HvBCAT-1* and the target, as well as the corresponding match on the gene structure of *HvBCAT-1,* are reported*.*

| **Genomic location *** | **Overlapping gene*** | **%ID** | ***HvBcat-1* exon** |
| --- | --- | --- | --- |
| 4A:24465877-24465914 | Traes_4AS_6C0AB5E18 | 92.1 | 1 |
| 4D:22280222-22280253 | Traes_4DL_9D7A18BBC | 93.8 |  |
| 4A:24466205-24466271 | Traes_4AS_6C0AB5E18 | 98.5 | 2 |
| 4D:22279863-22279929 | Traes_4DL_9D7A18BBC | 98.5 |  |
| 4A:24466438-24466803 | Traes_4AS_6C0AB5E18 | 96.2 | 3 |
| 4D:22279324-22279689 | Traes_4DL_9D7A18BBC | 95.4 |  |
| 4D:22278974-22279208 | Traes_4DL_9D7A18BBC | 97 | 4 |
| 4A:24466921-24467154 | Traes_4AS_6C0AB5E18 | 95.7 |  |
| 4A:24467240-24467384 | Traes_4AS_6C0AB5E18 | 95.9 | 5 |
| 4D:22278746-22278890 | Traes_4DL_9D7A18BBC | 95.9 |  |
| IWGSC_CSS_4BL_scaff_7034480:3-147 | Traes_4BL_FC8629A72 | 94.5 |  |
| 4A:24467551-24467667 | Traes_4AS_6C0AB5E18 | 96.6 | 6 |
| IWGSC_CSS_4BL_scaff_7034480:310-426 | Traes_4BL_FC8629A72 | 94.9 |  |
| 4D:222784479-22278595 | Traes_4DL_9D7A18BBC | 94.9 |  |
| IWGSC_CSS_4BL_scaff_7034480:310-426 | Traes_4BL_FC8629A72 | 89.4 | 7 |
| 4D:22278259-22278371 | Traes_4DL_9D7A18BBC | 89.4 |  |
| 4A:24467775-24467887 | Traes_4AS_6C0AB5E18 | 87.6 |  |

******Genomic location and the identification numbers of the overlapping genes refer to the IWGSC CSS assembly of bread wheat (Chinese Spring*

**Supplementary Table S4. Matches of the durum wheat genes *TdBCAT-*A and *TdBCAT-B* on the Ensembl Plants database.** Three different assemblies of *T. aestivum* CSS (IWGSC, 2014), TGAC v1 (Clavijo et al., 2017), and RefSeq v1 (IWGSC, 2018) were used to gain the complete *TdBCATs* sequence.

| **Gene** | **CSS** | **TGAC v.1** | **RefSeq v.1** |
| --- | --- | --- | --- |
| *TdBCAT-A* | Traes_4AS_6C0AB5E18 | TRIAE_CS42_4AS_TGACv1_307232_AA1018650 | TraesCS4A01G059800 |
| *TdBCAT-B* | Traes_4BL_FC8629A72 | TRIAE_CS42_4BL_TGACv1_321464_AA1060900 | TraesCS4B01G235400 |

**Supplementary Table S5**. Relative expression of *TdDHN15.3* and *TdBCAT* genes in spikes and flag leaves of 2 durum wheat cultivar at three different time-point: 7 DAT (acclimation to DS during anthesis), 11 DAT and 21 DAT during the early post-anthesis events. Values represent fold-change mean values. DAT= days after treatment.

|  | Colosseo | | | | | | Cappelli | | | | | |
| --- | --- | --- | --- | --- | --- | --- | --- | --- | --- | --- | --- | --- |
|  | Flag leaf | | | Spike | | | Flag leaf | | | Spike | | |
|  | 7 DAT | 11 DAT | 21 DAT | 7 DAT | 11 DAT | 21 DAT | 7 DAT | 11 DAT | 21 DAT | 7 DAT | 11 DAT | 21 DAT |
| *TdBCAT-A* | 2.07 | 0.88 | 2.40 | 0.51 | 4.62 | 0.69 | 5.64 | 9.95 | 13.50 | 1.50 | 0.82 | 2.42 |
| *TdBCAT-B* | 3.21 | 1.24 | 3.54 | 3.61 | 0.75 | 1.47 | 8.53 | 8.77 | 16.37 | 1.65 | 0.50 | 2.44 |
| *TdDHN15.3* | 0.63 | 1.87 | 2.55 | 1.39 | 4.40 | 1.17 | 19.84 | 9.52 | 7.73 | 8.63 | 3.81 | 4.63 |

**Supplementary Figure 1**. Multiple alignment of *Bcat* gene sequences: *TaBCAT-A* and *TaBCAT-B* cv. Chinese Spring, *TdBCAT-A* and *TdBCAT-B* cv. Svevo, cv. Cappelli and cv. Colosseo. *TdBCAT-B*. The coding regions are surrounded with red boxes. Cappelli and Colosseo share the same *TdBCAT-B* sequences, thus they are uniquely indicated as “*TdBCAT-B Cap&Col*”.
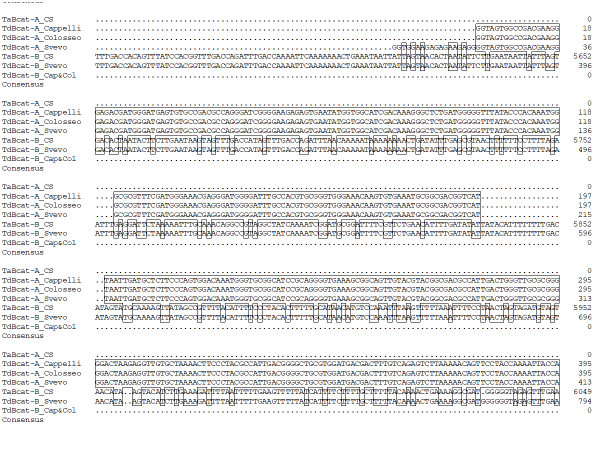

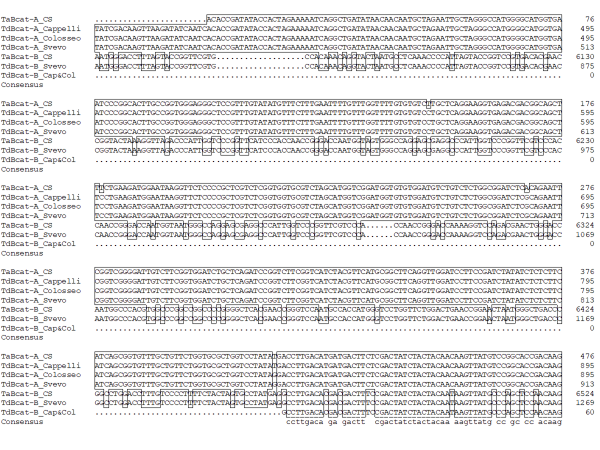


**
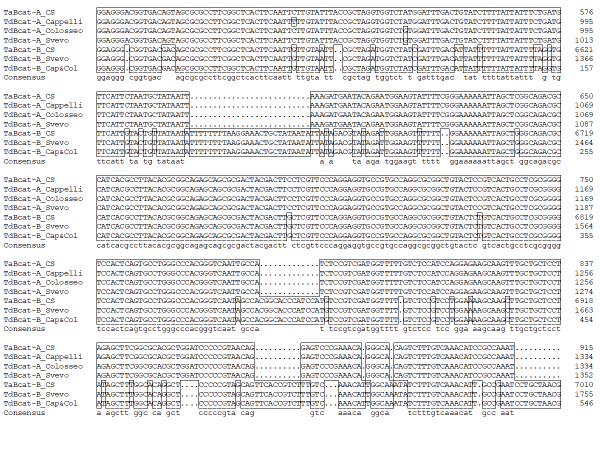

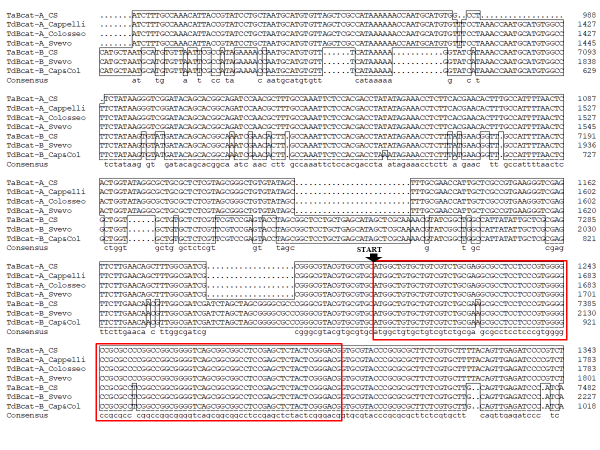
**

**
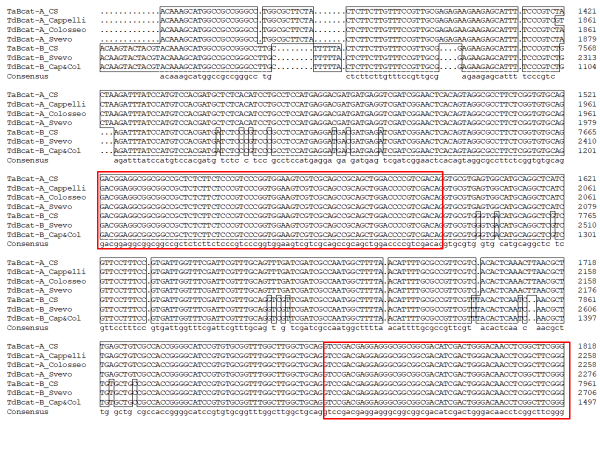

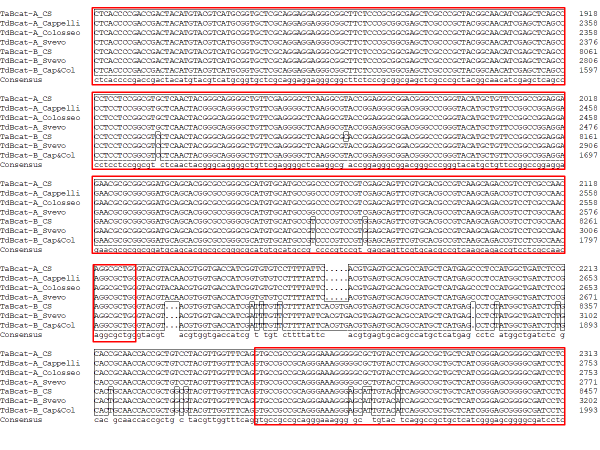
**

**
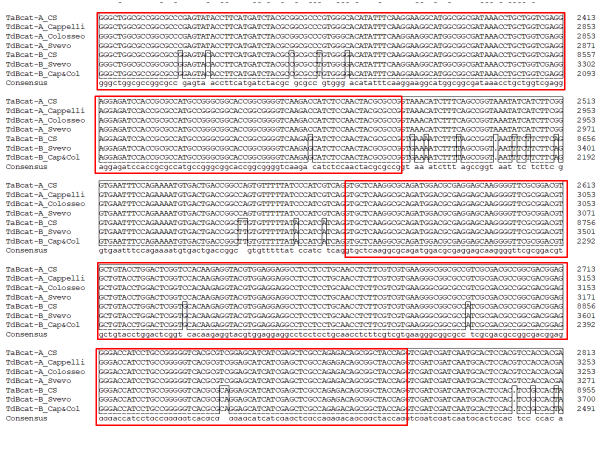

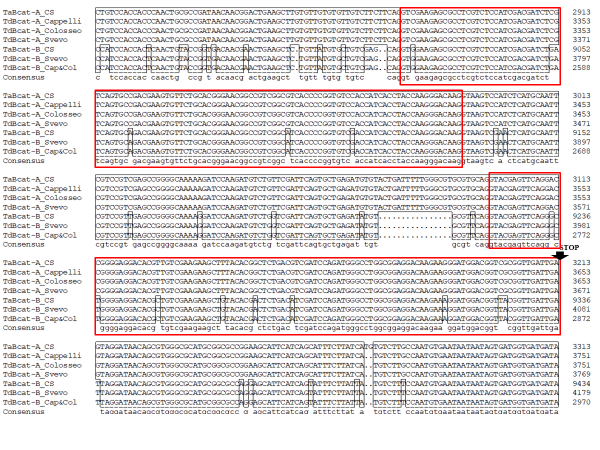
**

**
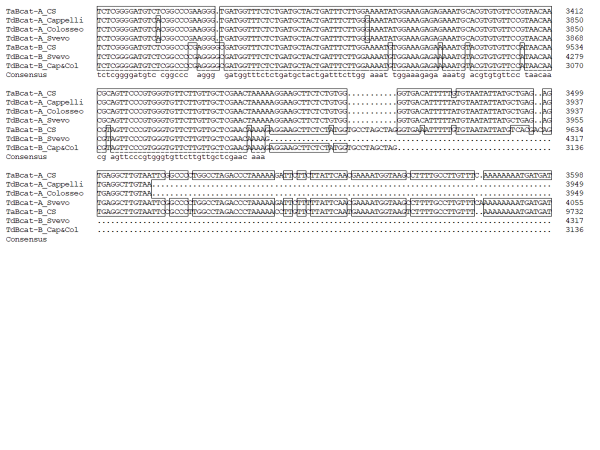
**
